# Factors associated with interruption of treatment among Pulmonary Tuberculosis patients in Plateau State, Nigeria. 2011

**DOI:** 10.11604/pamj.2014.17.78.3464

**Published:** 2014-01-31

**Authors:** Luka Mangveep Ibrahim, Idris Suleiman Hadejia, Patrick Nguku, Raymond Dankoli, Ndadilnasiya Endie Waziri, Moses Obiemen Akhimien, Samuel Ogiri, Akin Oyemakinde, Ibrahim Dalhatu, Okey Nwanyanwu, Peter Nsubuga

**Affiliations:** 1 Nigeria Field Epidemiology and Laboratory Training Program, Nigeria; 2 Department of Community Medicine, Ahmadu Bello University, Zaria, Nigeria; 3 World Health Organization, Nigeria; 4 Centers for Disease Control and Prevention, Nigeria Country Office, Abuja Nigeria; 5 Global Public Health Solution, Decatur GA USA

**Keywords:** Interruption, treatment, Tuberculosis, Nigeria

## Abstract

**Introduction:**

Nigeria has one of the highest tuberculosis (TB) burdens in the world with estimated incidence of 133 per 100,000 populations. Multi-drug resistant TB (MDR-TB) is an emerging threat of the TB control in Nigeria caused mainly by incomplete treatment. This study explored factors that affect adherence to treatment among patients undergoing direct observation of TB treatment in Plateau state, Nigeria.

**Methods:**

Between June and July 2011, we reviewed medical records and interviewed randomly selected pulmonary TB patients in their eighth month of treatment. Information on patients? clinical, socio-demographic and behavioral characteristics was collected using checklist and structured questionnaire for knowledge of treatment duration and reasons for interruption of treatment. We conducted focus group discussions with patients about barriers to treatment adherence. Data were analyzed with Epi Info software.

**Results:**

Of 378 records reviewed, 229 (61%) patients were male; mean age 37.6 ±13.5 years and 71 (19%) interrupted their treatment. Interruption of treatment was associated with living > 5 km from TB treatment site (AOR: 11.3; CI 95%: 5.7-22.2), lack of knowledge of duration of treatment (AOR: 6.1; CI 95%: 2.8-13.2) and cigarette smoking (AOR: 3.4; CI 95%: 1.5- 8.0). Major reasons for the interruption were lack of transport fare (40%) and feeling well (25%). Focused group discussions revealed unfriendly attitudes of health care workers as barriers to adherence to treatment.

**Conclusion:**

This study revealed knowledge of the patients on the duration of treatment, distance and health workers attitude as the major determinants of adherent to TB treatment. Training for health care workers on patient education was conducted during routine supportive supervision.

## Introduction

Tuberculosis (TB), a disease for which effective cure and preventive measures was discovered decades ago is still a major public health problem globally. It accounted for over a million deaths in 2010, 95% of the deaths occurring in low and middle income countries [1]. In 2011, Nigeria with an estimated incidence of 133/100,000 population ranked ninth among the 22 countries that account for 80% of the TB burden in the world [2].

Nigeria adopted the World Health Organization recommended Directly Observed Treatment Short Course (DOTS) for TB control since 1996 using the 8 months treatment regimen. Under the DOTS strategy patients are classified for treatment into category 1 for patients receiving TB treatment for the first time and category 2 for patients who had previous TB treatment. The DOTS strategy requires that patients swallow their drugs under the direct observation of the health care workers at least in the first 2 to 3 months of treatment [3]. This strategy prevents patients from interrupting their treatment and ensures they adhere to the treatment for the expected duration. Effective implementation of the strategy leads to elimination of the reservoir of the infectious agent. Interruption and non completion of treatment affects the outcome of the treatment and the overall performance of the TB control program. A TB patient with positive sputum for *Mycobacterium tuberculosis* who interrupts or fails to complete treatment is a potential source of multi-drug resistant TB (MDR-TB) [3]. MDR-TB is one of the emerging threats to the success of the TB control program in Nigeria. According to the national TB drug resistance survey, MDR-TB prevalence rates was 2.9% among new patients and 14.3% among patients who had previous exposure to anti TB drugs in 2012 [4].

Plateau state, one of the 36 states in Nigeria is located in the north central geopolitical zone of the country covering a land mass of 22,410 square kilometers (5) and had a population of 3.7 million people in 2010. TB control services using the DOTS strategy started in five health facilities in 2001 and in 2010 there were 198 health facilities offering TB treatment sites indicating geographical distribution of 1 treatment center per 113 square kilometer. Review of the outcomes of TB treatment in the state from 2001 to 2010 revealed that the highest cure rate of 63.5% was lower than the expected national and global target of at least 85% and the lowest default rate ever achieved of 6.5% was also higher than the 3% national target [3]. As a result of the low cure and high default rates, we conducted a study to determine the proportion of TB patients with interrupted treatment, to identify the factors associated with interruption of TB treatment. Interruption of treatment is one of the key determinant factors for outcome of treatment. The findings of the study will help the TB control program to focus interventions on causes of poor outcome of TB treatment and prevent the emergence of MDR-TB in the state.

## Methods

### Study setting

The study was conducted in Plateau state north central Nigeria between June and July 2011. We conducted a cross-sectional study among pulmonary TB patients registered for treatment at least 7 months before the commencement of the study. We included patient aged 15 years and above, diagnosed by sputum AFB microscopy, chest X-ray, or a clinician.

We defined TB treatment interruption as any TB patient who missed treatment for 2 consecutive days in the intensive phase of treatment (which is the first 2 months of treatment for category 1 and first 3 months for category 2 patients). It also applied to category 1 patient who missed 14 consecutive days and category 2 patients who missed 2 consecutive days in the continuation phase of treatment.

### Sample size, data collection and analysis

We calculated sample size using a power of 80% and 95% confidence interval. The highest cure rate of 63.5% achieved in 2006 was used as the proportion of interest with degree of precision of 0.05. Using n = z^2^xpq/d^2^gave us a minimum sample size of 392.

The TB central register which contained names of all patients diagnosed and registered for treatment was used to determine the sampling frame. Of the 798 patients eligible for the study, 392 were selected by systematic random sampling. The patients were traced and interviewed using an interviewer administered structured questionnaire. We extracted information on patients? social, economic and behavioral characteristics, knowledge on treatment duration, and factors associated with interruption of treatment. Information on the distance of the patients? place of abode to the health facilities was measured by the mileage of the vehicles (motorbikes and car) that we used to trace the patients.

We conducted focus group discussions with homogeneous groups (male and female groups) to eliminate cultural barriers that might affect active participation among the selected patients. The focus group discussions were conducted by a team of 2 research assistants, a note taker and a moderator. We used a pre-designed focus group discussion guide to ask questions on knowledge and factors responsible for interruption of TB treatment. The discussion was recorded.

We entered the data into Epi info version 3.3.2 database. We performed descriptive, bivariate, and multivariate analysis and odds ratios were used to compare categorical variables at 95% confidence intervals. The audio recorded focus group discussions were transcribed into written form, translated from the local language (Hausa) used in the discussion into English and analyzed according to the specific thematic areas and reported as narration.

### Ethical consideration

Ethical clearance was obtained from the Plateau State Health Research Ethics Committee. Informed consent was obtained from all respondents involved in the study.

## Results

A total of 378 patients were interviewed giving a response rate of 96% (378/392). 229 (61%) patients were male. The mean age of all patients was 37.6 years (standard deviation = 13.5 years). The mean ages for males was 38.5 years and for females was 35.8 years. 61% (230) were within the age brackets of 25-44 years (Table 1).

**Table 1:** Demographic characteristics among Pulmonary TB patients in Plateau state 2011

Age group in years	Sex		Total Frequency (%)
	FemaleFrequency (%)	MaleFrequency (%)	
15-24	21 (14)	27 (12)	48 (13)
25-34	60 (40)	77 (34)	137 (36)
35-44	39 (26)	54 (24)	93 (25)
45-54	13 (8.7)	53 (17)	53 (14)
> 54	16 (11)	31 (14)	47 (12)
Total	149 (39)	229 (61)	378 (100)

A total of 71 (19%) of the patients had an incident of interrupting their treatment (Table 2). Patients who interrupted their treatment gave long distance from TB treatment sites, feeling and lack of knowledge of the duration for the treatment as the major reasons for their actions. Seventy-two percent of the patients were currently married, 19% (70) gave history of cigarette smoking, 44% (167) gave history of alcohol use, and 19% (72) lived more than 5 km from their TB treatment sites

**Table 2:** Clinical, social and behavioral characteristics respondents among Pulmonary TB

Patients factors		Frequency	%
**Category 2 clinical class**	Yes	34	9.0
**Patient interrupted treatment**	Yes	71	19
**Marital status**	Married	271	72
	Single	81	21
	Separated	11	2.6
	Divorced	10	2.9
	Widow	5	1.3
**Occupation**	Farming	134	35
	Business	75	20
	Civil servants	64	17
	Applicant	45	12
	Schooling	38	10
	Others	22	5.8
**Educational levels**	None	69	18
	Primary	93	25
	Secondary	121	32
	Post-secondary	68	18
	Qur’ anic	27	7.1
**Patients role in the family**	Main wage earner	180	48
	Supports family	58	15
	House wife	80	21
	Dependent	60	16
**Distance from TB treatment sites**	Living > 5 km from site	72	19
**Cigarette smoking**	Ever smoked cigarette	70	19
	Currently smoke cigarette	16	4.2
**Alcohol use**	Ever used alcohol	167	44
	Currently use alcohol	61	16

Patients in Plateau state 2011 (n=378)

Distance of patients’ place of abode more than 5 km from TB treatment sites (adjusted odds ratio = (AOR): 11.3; CI 95%: 5.7 - 22.2), lack of knowledge of duration of treatment of TB (AOR= 6.1; CI 95%: 2.8 - 11.2) and cigarette smoking (AOR= 3.4; CI 95%: 1.5 - 8.0) remained independent determinants for interruption of treatment among the respondents (Table 2). Participants in the focus group discussions identified cost of transportation to the clinic for direct observation of treatment and unfriendly attitude of the health care workers as the major factors responsible for interruption of treatment. A male participant said? *they (health care workers) cause us more problems when they are harsh to us?*.

**Table 3:** Factors associated with interruption of treatment among Pulmonary TB patients in Plateau state 2011 (n = 378)

Factors	N/total	%	OR (95% CI)	AOR (95% CI)
Living > 5km from treatment site	38/72	19	9.3 (4.94-17.38)	11.3 (5.72-22.19)
Lack of knowledge of duration of treatment	23/58	15	3.7 (2.03-6.85)	6.1 (2.80-13.22)
History of cigarette smoking	20/70	19	2.0 (1.11-3.67)	3.4 (1.49-7.95)
Employment status (Unemployed)	22 /85	23	1.7 (0.98-3.09)	1.6 (0.66-3.70)
Category classification 2	8/34	9.0	1.4 (0.54-3.38)	2.1 (0.76-5.95)
Sex of patient (female)	27/149	39	1.1 (0.63-1.83)	1.4 (0.55-3.47)
Age > 35 years	35/176	47	1.1 (0.66-1.96)	0.7 (0.34-1.44)
≤ Primary Education	36/189	50	1.0 (0.62-1.73)	1.5 (0.77-3.05)
Main wage earner in family	32/180	48	0.9 (0.52-1.48)	0.7 (0.25-1.82)
History of alcohol use	28/167	44	0.8 (0.45-1.37)	0.6 (0.31-1.32)
Marital status (Married)	48/271	72	0.4 (0.44-1.43)	0.7 (0.31-1.69)

## Discussion

We found that 19% of the TB patients in Plateau state interrupted their treatment. The major factors associated with patients’ interrupting treatment were long distance of patients from the treatment sites, lack of knowledge of the duration of treatment, and cigarette smoking.

Access to TB treatment is one of the key determinants for effective TB control services. Distance may limit patient access to the services especially for the daily direct observation of the treatment (DOT) which takes place in the health facility during the intensive phase of treatment. Free TB treatment policy is being implemented in Nigeria with the main aim at reducing cost to the patient. However, the patients have to pay their travel cost to the clinic to access the service. In the state where the geographical distribution of the TB treatment facilities is as low as 1 health facilities per 113 square kilometer, this is further compounded by skewed distribution towards the urban areas making accessibility a great concern to patients in rural areas. These patients who are already impoverished by the disease are also faced additional costs for transporting themselves to access the TB services making them more likely to interrupt treatment. Our findings are in congruent with those reported by O'Boyle et al in Malaysia [6] and Kandel et al in South Africa [7] they reported long distance, costs of travel and travel time as the major risks factor for interruption of treatment among TB patients. TB treatment takes a long duration, in our case it takes 8 months. The disappearance of symptoms is an indication of clinical improvement from diseases and a measure of the effectiveness of the therapy. Because of the high quality drugs used in the DOTS strategy it is common place for TB symptoms to disappear even within a few weeks of treatment. Patients with inadequate knowledge of the duration of the treatment may feel that they are cured and thus stop the treatment. Kaona et al in their study on assessment of factors contributing to TB treatment adherence in Ndola, Zambia showed that feeling well was the major reason for patient stopping treatment [8]. Their finding is similar with our results which revealed that lack of knowledge of duration of treatment was significantly associated with interruption of treatment.

TB treatment involves a lot of interaction between patients and health care workers. Thus the attitude of the health care workers towards the patient remains important factor that can keep the patients on treatment or make them break the treatment or abandon it. Unfriendly attitude of health care workers might make patients feel threatened and unwelcomed leading to treatment interruption. The negative effect of untoward attitude of health care workers on TB treatment had been reported in India by Jaiswal et al. They noted that patient who defaulted from treatment blamed the health workers for their unpleasant behavior and attitude towards them whom they described as rude and unhelpful [9]. Patients who are treated with love and empathy by the health care workers will probably be more willing to stay on their treatment to completion.

The demographic characteristics and behavior of the patients have been shown to play a significant role in the occurrence of diseases including their behavior towards the treatment. The importance of age in patient adherence to treatment was reported by Wu et al in Taiwan. They reported that elderly patients had poorer treatment outcomes because they needed additional support to access TB treatment [10]. Similarly gender had been shown to influence the behavior of patient towards TB treatment. Cultural practices are one of the important factors with great influence on female health seeking behavior including adherence to TB treatment in many developing countries. Studies from Africa, Bangladesh and Syria showed that most married women must seek permission from their husband to attend health care services including TB treatment [11–13]. This might cause potential barrier to the TB treatment. Despites this barrier they tend to adhere to anti-TB treatment leading to better treatment outcome than men indicating that there could be hidden factor among the female. These findings contrast the results of our study which showed no significant relationship between age and gender and interruption of treatment. Furthermore, behavioral factors especially cigarette smoking and alcohol use have negative effect on TB treatment. Cigarette smoking is known to damage the lungs and suppresses the individual adaptive immune responses affecting patients? response to TB treatment [14–16]. Cigarette smoking had been shown to be associated with interruption with treatment similar to findings in Turkey [17] and India [18] although the mechanism is not well understood. Alcohol suppresses the immune response. Also, alcoholics are more likely to forget taking their treatment and hospital appointments leading to interruption. Its use and non adherence had been reported by many studies [19–21]. Our study did not show significant relationship with interruption of treatment despite the high proportion of our respondents who reported alcohol use.

Our study had limitation on information on distance, cigarette smoking and alcohol use. Our data were based on the geographical distance. We did not explore the means of transport, the travel time, and cost of transport from the patient's home to the clinic. These are important predictive factors for non-adherence to treatment. Our data on cigarette smoking and alcohol use were based on self-reporting by the respondents it is possible that some patients could have concealed their cigarette smoking status and alcohol use. The application of biomarkers to test for these substances in the respondents? skin or body fluid could have given more definitive information on their status. Furthermore, we did not explore from the health care workers? factors that could motivate them to relate well with their patients. Finally, we did not segregate our data on the method used for diagnosis of the patients. Patients? adherence to treatment might be affected by presence of convincing evidence of the presence of the disease. Patients diagnosed by X-ray or clinician are likely to interrupt their treatment because of the lack of this objective evidence.

## Conclusion

Interruption of TB treatment in Plateau state is associated with long distance from treatment sites including cost of transport, poor knowledge of duration of TB treatment, cigarette smoking, and unfriendly attitude of health care workers towards patients. Based on our findings, supportive supervision was initiated with training of health workers on patients? education on duration of treatment and the danger of interruption of treatment. We have also begun decentralization of TB treatment sites and use of patients? relations or community members to support and encourage them on their treatment and to observe the daily intake of the drugs by the patients at home to improve accessibility of services to patients.

## Notes

**Recommendations:** The Government of Plateau state should support and sustain the decentralization process including the implementation of community TB cares. The government of Nigeria and the National TB control program should support and promote the involvement of all eligible private health care providers under the Public-Private Mix directly observed treatment short course strategy (PPM-DOTS) to ensure availability and accessibility of the services to patients. The National TB Training Center in Nigeria should include training of TB control service providers on inter personal communication to improve relationship between patient and health care providers.
